# Direct Neuronal Reprogramming Reveals Unknown Functions for Known Transcription Factors

**DOI:** 10.3389/fnins.2019.00283

**Published:** 2019-03-26

**Authors:** Gaia Colasante, Alicia Rubio, Luca Massimino, Vania Broccoli

**Affiliations:** ^1^Stem Cell and Neurogenesis Unit, Division of Neuroscience, San Raffaele Scientific Institute, Milan, Italy; ^2^CNR Institute of Neuroscience, Milan, Italy

**Keywords:** stem cells, cell reprogramming, neuronal differentiation, brain development, transcription factor

## Abstract

In recent years, the need to derive sources of specialized cell types to be employed for cell replacement therapies and modeling studies has triggered a fast acceleration of novel cell reprogramming methods. In particular, in neuroscience, a number of protocols for the efficient differentiation of somatic or pluripotent stem cells have been established to obtain a renewable source of different neuronal cell types. Alternatively, several neuronal populations have been generated through direct reprogramming/transdifferentiation, which concerns the conversion of fully differentiated somatic cells into induced neurons. This is achieved through the forced expression of selected transcription factors (TFs) in the donor cell population. The reprogramming cocktail is chosen after an accurate screening process involving lists of TFs enriched into desired cell lineages. In some instances, this type of studies has revealed the crucial role of TFs whose function in the differentiation of a given specific cell type had been neglected or underestimated. Herein, we will speculate on how the *in vitro* studies have served to better understand physiological mechanisms of neuronal development *in vivo*.

## Introduction

Over the years, crucial extrinsic and intrinsic mechanisms regulating the acquisition of cell fate during neural development have been elucidated. Gradients of morphogens secreted by organizer centers instruct neural progenitor cells (NPCs) to activate the expression of transcription factor (TF) cascades that guide cells through every single step of the fate acquisition process. Genetic studies *in vitro* and *in vivo*, essentially based on the gain- and loss-of-function experiments, revealed that large arrays of TF cascades are indeed responsible for the specification of different neuronal subtypes.

This mechanistic knowledge was critical for the field of cell reprogramming to emerge. Indeed, the possibility to convert a cell type into another has been strictly dependent on seminal findings accumulated over the last 30 years in neurodevelopmental biology.

Back in the 1950s, it was not yet clear whether all cells belonging to the same organism contained the same set of genes. On this line, Weismann had suggested that genes whose function was no longer required might be lost or permanently inactivated in a specific cell type, seeding the concept that cell fate acquisition is an irreversible process being associated with loss of genetic material. This concept, well represented by the famous Waddington’s landscape (Waddington, 1957) was later challenged by Gurdon’s work. He performed pioneer experiments of somatic nuclei transfer in *Xenopus* oocytes during his Ph.D. studies, providing the first evidence for the preservation of genome integrity after cellular differentiation (Gurdon, 1962).

Up to date, it is consolidated the concept that epigenetic-mediated gene silencing, rather than gene loss, accompanies cell fate acquisition. This evidence opened a crack toward the plasticity of cell identity and the possibility of altering the fate of a differentiated cell.

In 1988, *MyoD* ectopic expression in mouse embryonic fibroblasts (MEFs) was revealed sufficient to convert them into muscle cells (Tapscott et al., 1988). Two decades later the breakthrough from the Yamanaka’s group showed that somatic cells can be reverted to a pluripotent state forcing the expression of the four factors *Oct4, Sox2, Klf4*, and *c-Myc* (Takahashi and Yamanaka, 2006), that are mediating global chromatin remodeling allowing for the expression of the pluripotency gene machinery (Takahashi and Yamanaka, 2006; Boissart et al., 2012). First successful conversion of MEFs into functional induced neurons (iNs) was described a few years later through *Ascl1*, *Brn2*, and *Myt1l* misexpression (Vierbuchen et al., 2010). After this study, many others attempted to modify or enrich this TF combination to induce MEF differentiation toward specific neuronal subtypes localized to defined areas of the brain (reviewed in Masserdotti et al., 2016).

All these works highlighted the ability of accurately selected cocktail of TFs to alter the fate of fully differentiated cells and to obtain functional neuronal cells.

To define a cell reprogramming gene cocktail, the TFs to be tested in the screening are chosen for their capability to impose that specific neuronal fate (master regulator genes) or among genes enriched in the target cell population, but not necessarily with their functions already addressed. Very recently, unbiased screenings of TFs for neuronal conversion have been also performed with very informative results (Liu et al., 2018; Tsunemoto et al., 2018). Once the candidate TF list is selected, they are delivered in donor cells according to a “narrow down” or an “add one” strategy. Generally, TFs are delivered and expressed all simultaneously in the donor cells although they control different phases of the cell fate acquisition process. In other cases, genetic tricks (i.e., mix of constitutive promoter guided- and inducible promoter guided-TFs whose expression can be turned off at a defined time) are employed to allow sequential expression of TFs required in different phases of differentiation in a manner that tries to recapitulate the expression timing observed during *in vivo* development (Au et al., 2013; Colasante et al., 2015). Finally, in an even more sophisticated experimental setting, the endogenous loci of the desired TFs can be activated using the CRISPR/Cas9 system (Black et al., 2016; Liu et al., 2018). In all these cases, the final output of these studies can meet the initial expectations, but unpredicted results have not rarely been reported. In fact, in some instances, new features for the mechanisms of action of TFs have been emerging. In others, TFs whose role was not considered determining for a specific neuronal fate acquisition during *in vivo* development, have come out as pivotal in the neuronal specification during direct cell reprogramming. Even more surprisingly is the identification of TFs not related to neuronal development that are able to impose a neuronal identity when overexpressed in heterologous cells.

This predictive value of the direct cell reprogramming methodology can be likely explained by the fact that during this process selected TFs are forced to operate in donor cell populations that are very distant from the target neuronal cells. This is the case for the fibroblast-to-neuron conversion, as fibroblasts have a mesodermic origin in the embryo contrary to the ectoderm-derived neurons. According to this different ontogeny, fibroblasts present both divergent global gene expression profiles and chromatin states compared to neurons. In this “unfavorable environment,” some neuronal TFs unexpectedly revealed to have a pioneer function being able to “open up” the chromatin and activate genes that are silenced in donor cells. Conversely, *in vivo*, their function might be facilitated by other TFs expressed earlier in the transcriptional cascades or their function might be hidden by complex gene regulation networks. With its ability to directly challenge TFs, the direct neuronal reprogramming provides a unique experimental system where to better appreciate their role in a relatively simple *in vitro* assay with a clear phenotypic analysis outcome.

## New Insights Into the Roles of the Proneural TFs

### Deepening Our Understanding of Classical Proneural TFs: *Ascl1* and *Neurog2*

Textbook developmental biology studies revealed that Achaete-scute homolog 1 (*Ascl1*) and Neurogenin2 (*Neurog2*) are the prominent pro-neural factors in charge of the neuronal identity specification in the nervous system (Horton et al., 1999; Bertrand et al., 2002; Parras et al., 2002; Mattar et al., 2004; Schuurmans et al., 2004; Britz et al., 2006; Poitras et al., 2007; Kovach et al., 2013). These two TFs are expressed in a complementary manner in the telencephalon: *Neurog*2 is expressed in dorsal progenitors and instruct them to generate glutamatergic neurons, whereas *Ascl1* is expressed in ventral progenitor cells contributing to the acquisition of GABAergic fate.

With this well-established background, it seemed pretty consistent that the forced expression of *Ascl1* was shown essential to obtain neurons from both murine and human fibroblasts (Vierbuchen et al., 2010; Caiazzo et al., 2011; Kim et al., 2011; Pfisterer et al., 2011; Torper et al., 2013; Colasante et al., 2015; Table 1). The relevant role of *Ascl1* has also been demonstrated in the reprogramming of other cell types that are more plastic than terminally differentiated fibroblasts or more closely related to neurons. Indeed, *Ascl1* alone can guide the conversion of murine or human embryonic stem cells (ESCs) (Chanda et al., 2014) and astrocytes (Berninger et al., 2007; Heinrich et al., 2010; Liu et al., 2015; Masserdotti et al., 2015; Chouchane et al., 2017) into neurons (Table 1).

**Table 1:** Summary of the TF combinations that include *Ascl1* or *Ngn2* to directly reprogram somatic or pluripotent cells into specific iN subtypes.

Factors	Source	iN main subtype	Reference
Ascl1, Brn2, Myt1l	Fibroblasts	GABA/Gluta	Vierbuchen et al., 2010; Pfisterer et al., 2011
Ascl1, Myt1l, NeuroD2, miR-9/9^∗^, miR-124	Fibroblasts	GABA/Gluta	Yoo et al., 2011
Ascl1	Fibroblasts	GABA/Gluta	Chanda et al., 2014
Ascl1, Brn2, Myt1l	Fibroblasts	GABA/Gluta	Pereira et al., 2014
Ascl1, Brn2, Myt1l, Neurod1	Fibroblasts	Gluta	Pang et al., 2011
Ascl1, Sox2, FoxG1, Dlx5, Lhx6	Fibroblasts	GABA	Colasante et al., 2015
Ascl1, Brn2, Myt1l,Ngn2, Lhx3,Isl1, Hb9 (NeuroD1)	Fibroblasts	Motor	Son et al., 2011
Ascl1, Brn2, Myt1l, Lmx1a, Foxa2	Fibroblasts	Dopaminergic	Pfisterer et al., 2011
Ascl1, Nurr1, Lmx1a	Fibroblasts	Dopaminergic	Caiazzo et al., 2011
Ascl1, Pitx3	Fibroblasts	Dopaminergic	Kim et al., 2011
Ascl1, Brn2, Myt1l, Lmx1a, Lmx1b, FoxA2, Otx2	Fibroblasts	Dopaminergic	Torper et al., 2013
Ascl1, Dlx2	Astrocytes	GABA	Heinrich et al., 2010
Ascl1	Astrocytes	GABA	Chouchane et al., 2017
Ascl1, Nurr1, Lmx1b	Astrocytes	Dopaminergic	Addis et al., 2011
Ascl1	ESCs	GABA/Gluta	Chanda et al., 2014
Ascl1, Sox2, FoxG1, Dlx5, Lhx6	iPSCs	GABA	Colasante et al., 2015
Ascl1, Dlx2	ESCs, iPSCs	GABA	Yang et al., 2017
Ngn2, Ascl1	Fibroblasts	Gluta	Ladewig et al., 2012
Ngn2, Sox11, Isl1, Lhx3	Fibroblasts	Motor	Liu et al., 2015
Ngn2, Brn3a	Fibroblasts	Sensory	Blanchard et al., 2015
Ngn2, (Sox11)	Fibroblasts	Cholinergic	Liu et al., 2013; Smith et al., 2016
Ngn2	Astrocytes	Gluta	Heinrich et al., 2010; Chouchane et al., 2017
Ngn2, Bcl2	Astrocytes	Gluta	Gascón et al., 2016
Ngn2	Cerebellar Astrocytes	GABA	Chouchane et al., 2017
Ngn2	NPC	Gluta	Ho et al., 2016; Orellana et al., 2016
Ngn2	iPS, ESCs	Gluta	Zhang et al., 2013; Busskamp et al., 2014; Rubio et al., 2016


Very soon in the field, an amazing difference in the ability in fibroblast-to-neuron conversion was observed between *Ascl1* and its glutamatergic *alter ego*, *Neurog2*. Indeed, several studies indicated that the induction of *Neurog2* cannot reprogram fibroblasts efficiently while it can generate neurons when overexpressed in ESCs, induced pluripotent stem cells (iPSCs), NPCs and astrocytes (Berninger et al., 2007; Heinrich et al., 2010; Vierbuchen et al., 2010; Liu et al., 2013; Zhang et al., 2013; Busskamp et al., 2014; Chanda et al., 2014; Masserdotti et al., 2015; Ho et al., 2016; Orellana et al., 2016; Rubio et al., 2016; Table 1). As expected, in most of these cases the neurons acquired a glutamatergic identity. Only when it is induced in murine astrocytes of cerebellar origin, Neurog2 promotes the generation of GABAergic neurons in according to its role during embryo development where it drives the differentiation of GABAergic Purkinje cells (Florio et al., 2012; Chouchane et al., 2017).

The poor efficiency of *Neurog2* in the fibroblast-to-neuron conversion can be raised dramatically when *Neurog2* is expressed together with other transcriptional factors and/or in the presence of small molecules in the media (Son et al., 2011; Liu et al., 2012, 2013, 2015, 2016; Aravantinou-Fatorou et al., 2015; Blanchard et al., 2015).

**FIGURE 1:**
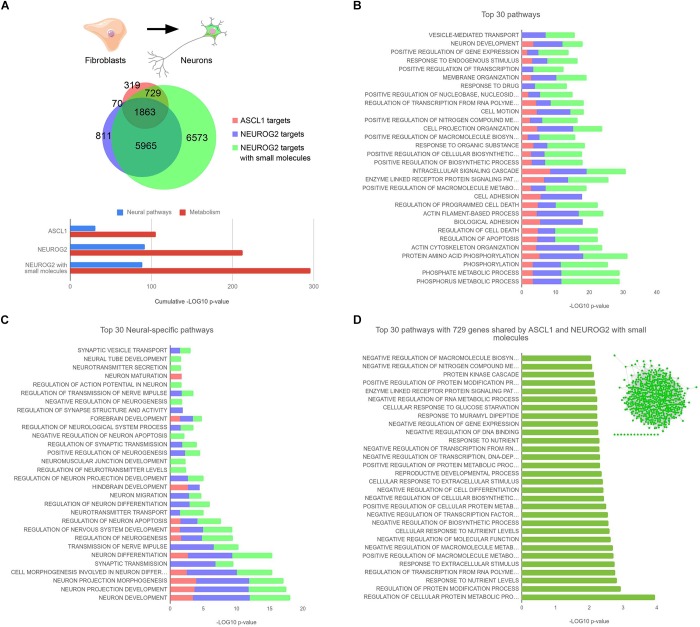
**(A)** Area-proportional Venn diagram depicting the total number of ASCL1 and NEUROG2 targets. The proportion of shared targets for the 3 experimental conditions (ASCL1, NEUROG2, and NEUROG2 with small molecules) are indicated. Bottom, result summary showing the cumulative enrichment of neural or metabolic pathways in the 3 conditions. **(B)** Top 30 pathways enriched in the 3 experimental conditions. **(C)** Top 30 neural-specific pathways in the 3 experimental conditions. **(D)** Top 30 pathways of the 729 genes shared by ASCL1 and NEUROG2 with small molecules. Inset: gene network generated with the GeneMania Cytoscape plugin, built in accordance with Physical interactions, Co-expression, Co-localization, corresponding Pathway, and Genetic interactions showing one single molecular hub of 715 genes, with 636 edges and a clustering coefficient of 0.135. ChIP-seq peaks were downloaded from NCBI GEO (GSE43916, GSE63621, and GSE75912) and annotated with annotatePeaks (Heinz et al., 2010). Functional enrichment analysis was performed with DAVID (Huang da et al., 2009). Filtering, statistics, and plotting were performed within the R environment.

We tried to clarify this intriguing difference between *Ascl1* and *Neurog2* in reprogramming efficiency of fibroblasts comparing side by side their direct molecular targets. To this aim, we took advantage of chromatin immunoprecipitation-sequencing (ChIP-seq) data already available in the literature. Smith et al. (2016) transduced MRC-5 human fetal fibroblasts with lentiviruses expressing either *ASCL1*, *NEUROG2*, or *NEUROG2* together with the small molecules forskolin and dorsomorphin to increase the reprogramming efficiency. Then, ChIP-seq analyses were performed at 2.5, 3, and 4 days after the infection for each condition and we cross-referenced the datasets merging all time points together (Figure 1). Focusing our attention on the conditions where (i) *ASCL1* or (ii) *NEUROG2* were induced, we found that *ASCL1* and *NEUROG2* share a consistent number of direct targets (1863) although maintaining many other exclusive (319+729 for ASCL1 and 811+5965 for NEUROG2) (Figure 1A).

Surprisingly, NEUROG2 targets are about nine times more abundant than those of ASCL1. Considering the difference in the reprogramming efficiency of these two TFs, we hypothesized that ASCL1 might be more efficient in the neuronal program activation by binding mainly neural genes among its targets. However, when we analyzed more deeply the top 30 GO (gene ontology) pathways targeted by *ASCL1*, we realized that most of them correlate with the activation of non-neural specific genes, i.e., GO related to alterations in intracellular pathways and metabolic changes (Figure 1B). Very few of them were instead related to neural differentiation program, for example, neuronal development (GO 0048666) and alterations in the cytoskeleton and plasma membrane (GO 0016044, GO 0007155, GO 0030029, and GO 0030036). Interestingly, the top 30 targets of *NEUROG2* were classified in GO categories similar to the ones observed in the case of *ASCL1*. When we focused on the Top 30 neural-specific pathways, it clearly emerged that *NEUROG2* exhibited even a higher enrichment of genes promoting neuralization in comparison to *ASCL1* (Figure 1C), not confirming our initial hypothesis.

To add more information to this picture, we analyzed also ChIP-seq datasets of NEUROG2 in presence of small molecules: in this condition, the access of *NEUROG2* to chromatin is even more enhanced likely due to the chromatin remodeling mediated by the small molecules possibly through the activation of SOX4 (Smith et al., 2016). In particular, in the presence of forskolin and dorsomorphin, NEUROG2 is able to bind to 729 novel genes that in the previous comparison were exclusive targets of ASCL1 (Figure 1A). Supposing that they contain the key genes responsible for the success of the reprogramming, we analyzed them more accurately. Again, we observed that these shared target genes do not belong mainly to neural categories (Figure 1D) suggesting that the activation of non-neural pathways, more than a prompt neuralization, is essential for the neuronal reprogramming in a “non-neuronal” context. Interestingly, we observe that most of these genes (715 out of 729) were either co-expressed, physically interacting, or belonging to the same molecular pathway, thus giving rise to a unique molecular network (Figure 1D, inset). This indicates that the activation of key regulatory genes that are connected to each other might guide the efficient conversion of fibroblasts into neurons. We also observed that 6573 targets are exclusive to the condition where the fibroblasts were treated with *NEUROG2* and the small molecules (Figure 1A). Since NEUROG2 in the presence of small molecules generates cholinergic neurons, differently from *ASCL1*, we believe that these genes or at least part of them might be involved in promoting the cholinergic fate. Alternatively, we cannot exclude that those targets themselves might be responsible for the acquired success of *NEUROG2* in the neuronal conversion process. In this case, the scenario would be different and would suggest that *NEUROG2* plus small molecules and *ASCL1* activate complementary gene regulatory networks that can independently reprogram fibroblasts into neurons. Further analysis is warranted to address this hypothesis.

Importantly, the prompt and massive neuralizing action of *NEUROG2* may be beneficial for the reprogramming of cells that are more closely related to neurons (such as astrocytes) or more prone to differentiate into neurons (such as ESCs, iPSCs, or NPCs). Indeed, in agreement with this speculation and assuming that the occupancy profiles would be similar using other cells, it has been reported that *NEUROG2*, when overexpressed in human ESCs, generates mature neurons faster than *ASCL1* (Chanda et al., 2014).

In synthesis, direct reprogramming enriched the classic knowledge on proneural genes highlighting different dynamics and kinetics in neural conversion mediated by *Ascl1* and *Neurog2* (summarized in Figure 1A, bottom). Those differences can underlie their different neuronal reprogramming efficiencies in different cellular lineages.

### The Emerging Function of the Previously Overlooked Proneural TF Myt1l

In other studies, direct cell reprogramming uncovered the importance during neuronal differentiation of TFs whose roles remained overlooked or underestimated during embryonic development. This is the case of Myt1l, encoding for a member of the zinc finger superfamily of TFs. Little work was performed in the past to understand the role of Myt1l in neural development until it emerged as an important factor in direct reprogramming (Vierbuchen et al., 2010). Since Myt1l is specifically expressed in neurons (Kim et al., 1997; Matsushita et al., 2014) it was selected together with other 18 genes as candidates to achieve fibroblast-to-neuron conversion. After careful screening, the authors defined that the minimal cocktail of 3TF necessary to reprogram fibroblasts included Myt1l indicating its importance, previously neglected, in neuronal development. Since then, Myt1l has been used in numerous protocols to reprogram non-neuronal cells into neurons (Ambasudhan et al., 2011; Marro et al., 2011; Pang et al., 2011; Pfisterer et al., 2011; Son et al., 2011; Yoo et al., 2011; Torper et al., 2013; Victor et al., 2014; Wainger et al., 2015). The experimental evidence indicate that Myt1l alone is not sufficient to obtain neurons, but it improves instead the efficiency of conversion and the morphology of the neurons when used together with *Ascl1* or miRNA (for example, in MEF (Vierbuchen et al., 2010), in human ES (Pang et al., 2011), in human fibroblasts (Victor et al., 2014). The discovery of this important role for Myt1l sparked the scientific community interest to better determine its mechanism of action during reprogramming but also *in vivo* during development. Studies in the reprogramming context have demonstrated that Myt1l is not a pioneer factor since it binds mainly open and active chromatin (Wapinski et al., 2013; Mall et al., 2017). In contrast, Myt1l acts as a transcriptional repressor that downregulates different cascade of non-neuronal genes, such as Notch and Wnt pathway, to promote neurogenesis (Mall et al., 2017). Importantly, Notch repression has been also confirmed in a physiological context for both Myt1l and Myt1, a gene highly homologous to Myt1l (Vasconcelos et al., 2016; Mall et al., 2017). Other data indicate the importance of Myt1l in neuronal development: Myt1l overexpression in NSCs and *in vivo* increases neuronal differentiation (Mall et al., 2017) and mutations in Myt1l have been associated with intellectual disability, schizophrenia and autism (Li et al., 2012; De Rubeis et al., 2014; De Rocker et al., 2015).

## Defining Gene Networks Responsible for Neuronal Subtype Specification

Direct reprogramming assays have further contributed to better tracking the functional interactions among TFs during the commitment of specific neuronal subtypes.

The discovery that *Ascl1* alone or with *Brn2* and *Myt1l* is able to generate glutamatergic iN (Vierbuchen et al., 2010; Chanda et al., 2014) and that, associated with other TFs, it seems necessary to generate every type of neurons (Caiazzo et al., 2011; Kim et al., 2011; Pfisterer et al., 2011; Torper et al., 2013; Colasante et al., 2015), resulted in direct contradiction with the undebated role of *Ascl1* during development in the specification of GABAergic neurons. Indeed, *Ascl1* was clearly described as an activator of *Dlx1/2* (Casarosa et al., 1999; Yun et al., 2002) and its ectopic expression in cortical ventricular zone (VZ) is sufficient to upregulate *Dlx1/2* (Fode et al., 2000), which in turn activates GAD65/67 expression (Stuhmer et al., 2002).

Five years later the first iN derivation, Colasante et al. (2015) suggested an answer to this conundrum revealing that *Ascl1* is effective in activating the GABAergic reporter GAD67-GFP during MEF to neuron conversion only if combined with either *Sox2* or *Foxg1*. Although their role in regulating the competence of telencephalic progenitors to adopt subpallial fates had been recently proposed (Manuel et al., 2010; Ferri et al., 2013), Colasante and colleagues, going deeper on the mechanism, showed that *Ascl1*, *Sox2*, and *Foxg1* strictly cooperate in determining the GABAergic fate. They showed that SOX2 and ASCL1- but not FOXG1-interact to bind and activate *Dlx1/2* enhancer, but the binding is allowed only when FOXG1 is also expressed. They hypothesized a chromatin pioneer role for FOXG1 that similarly to other forkhead-box TFs (Watts et al., 2011; Iwafuchi-Doi and Zaret, 2014), might open the repressed chromatin to enable SOX2 and *ASCL1* binding to the *Dlx1/2* locus.

Interestingly, cooperation between these three factors for the determination of a GABAergic fate was confirmed also *in vivo*. Cortical VZ cells express already *Sox2* and *Foxg1*, for this reason, it is sufficient to express *Ascl1* to allow a fate switch from glutamatergic to GABAergic (Fode et al., 2000). Conversely, the silencing of either *Foxg1* or *Sox2* in cortical VZ is sufficient to abolish the ability of *Ascl1* to induce a GABAergic neuronal fate when overexpressed in the same compartment. In accordance with this report, astrocytes over-expressing *Ascl1* can be converted in GABAergic neurons as they already express *Sox2* and *FoxG1* endogenously (Heinrich et al., 2010; Zhang et al., 2013; Masserdotti et al., 2015). The direct link between *FOXG1* and GABAergic fate emerged also when Mariani et al. (2015) observed that overexpression of the TF FOXG1 is responsible for the overproduction of GABAergic neurons in brain organoids modeling of autism spectrum disorders.

## Unexpected TFs Regulating Neuronal Reprogramming

Some recent works in the reprogramming field have contributed to identify TFs not previously related to neurogenesis that were unexpectedly able to generate differentiated neurons. The large majority of them has emerged by carrying out unbiased screenings of TFs able to produce neurons (Liu et al., 2018; Tsunemoto et al., 2018). In both these studies, the screening is based on systematic combinatorial strategies that are not exclusively relying on testing TFs differentially expressed between starting cells and desired target cells. Tsunemoto et al. (2018) tested 598 pairs of TFs cloned in doxycycline-inducible lentiviruses. They infected MEFs and observed which TF pair generated neurons that were functional. Surprisingly some of the identified TFs that could convert MEFs into neurons have never been related to the generation of neurons before, such as: OCT4, a well-known factor for cell pluripotency; Myf5, known mostly as a regulator of myogenesis; and Pit1, an anterior pituitary-specific TF. The screening also identified Ptf1a (Pancreas TF-1a), a TF that has been recently shown to generate NSCs when overexpressed in MEFs (Xiao et al., 2018).

Liu et al. (2018) performed an activatory CRISPR screening in mouse ESCs to systematically identify regulators of neuronal-fate specification. Among the top 20 TFs or DNA binding proteins most efficient in neuronal conversion, some of them (Ezh2, Suz12, Maz, Nr3c1, and Sin3b) were not preferentially enriched in neural cells as no differential expression was observed between the obtained neurons and mESCs. Ezh2 and Suz12 are two Polycomb-group proteins that act as global epigenetic regulators (Margueron and Reinberg, 2011) and can promote neuronal differentiation. Transcriptomic analyses suggested that Ezh2, in particular, acts mainly by inhibiting alternative endodermal and mesodermal lineages. Regarding the TFs not related with the neuronal identity the specific mechanism of action is yet to be defined. One plausible possibility is that, when expressed at supra-physiological levels, they are able to activate overlapping genetic pathways by ectopic binding to the same transcriptional targets through their sequence homology with their related neurogenic factors. Alternatively, they may have a yet unknown role during only a short window of time along the whole process of neuronal differentiation. Additional work is needed to fully investigate these alternative scenarios.

## Conclusion

Direct cell reprogramming proved to be informative in defining the dynamics and specific roles for several TFs and clarifying their molecular functions in yet unexplored gene networks. More unbiased screenings of TFs for neuronal conversion should be pursued (Liu et al., 2018; Tsunemoto et al., 2018), since they can help to better define these networks identifying remaining TFs not yet related to neuronal development but that can facilitate cell conversion. Finally, cell reprogramming studies generated a renewed interest in better deciphering TF functions during development, stimulating new studies *in vivo.* In overall, direct cell reprogramming can be considered as the first stage where newly TFs or unprecedented functions of well-known TFs can make their debut.

## Data Availability

All datasets generated for this study are included in the manuscript and/or the supplementary files.

## Author Contributions

LM performed computational analysis. GC, AR, and VB wrote the manuscript.

## Conflict of Interest Statement

The authors declare that the research was conducted in the absence of any commercial or financial relationships that could be construed as a potential conflict of interest.
